# NLRC5 Inhibits Inflammation of Secretory Phase Ectopic Endometrial Stromal Cells by Up-Regulating Autophagy in Ovarian Endometriosis

**DOI:** 10.3389/fphar.2020.01281

**Published:** 2020-08-18

**Authors:** Runhua He, Xiaojing Liu, Jing Zhang, Zhongzheng Wang, Wenyan Wang, Liutao Fu, Yijun Fan, Shiying Sun, Yunxia Cao, Lei Zhan, Lijun Shui

**Affiliations:** ^1^ Department of Gynecology and Obstetrics, The Second Affiliated Hospital of Anhui Medical University, Hefei, China; ^2^ Reproductive Medicine Center, Department of Obstetrics and Gynecology, the First Affiliated Hospital of Anhui Medical University, Hefei, China; ^3^ Clinical Center of Reproductive Medicine, The First Affiliated Hospital of USTC, Division of Life Science and Medicine, University of Science and Technology of China, Hefei, China

**Keywords:** NLRC5, ovarian endometriosis, ectopic endometrial stromal cells, autophagy, inflammation

## Abstract

Nod-like receptor (NLR) family caspase activation and recruitment domain containing 5 (NLRC5) is a newly identified sub-class of the NLR family. It regulates inflammation and has a key function in innate and adaptive immunologic reactions. Autophagy has been reported to be crucially linked to the pathogenesis of endometriosis. Our recent study identify there is a negative correlation between NLRC5 and autophagy in endometriosis, indicating that NLRC5 and autophagy together act as promising predictors in endometriosis patients. However, the mechanism associating NLRC5 and autophagy in endometriosis is still not completely understood. We hypothesize that autophagy could be involved in NLRC5-mediated inflammation in endometriosis. In order to validate the assumption, we evaluate the effects of NLRC5 and autophagy in the inflammation of ectopic endometrial stromal cells (EESCs) of ovarian endometriosis patients, to specifically determine whether autophagy is involved in NLRC5-mediated inflammation in EESCs. Our results show that over-expression of NLRC5 results in the up-regulation of autophagy in EESCs and inhibition of NLRC5 restricts the level of autophagy in EESCs. Furthermore, over-expression of NLRC5 and promotion of autophagy inhibit interleukin-6 (IL-6) and tumor necrosis factor-α (TNF-α) expressions, whereas inhibition of NLRC5 and autophagy up-regulate IL-6 and TNF-α expressions in EESCs. Additionally, promotion of autophagy contributes to the NLRC5-mediated inhibition of IL-6 and TNF-α expressions in EESCs; inhibition of autophagy restricts NLRC5-mediated inhibition of IL-6 and TNF-α expressions in EESCs. Our results suggest that over-expression of NLRC5 promotes autophagy, thereby inhibiting inflammation in ovarian endometriosis.

## Introduction

Endometriosis is a benign gynecologic disorder defined as the ectopic growth of endometrium outside the uterus, on the ovaries, pelvic peritoneum, and rectovaginal septum. Endometriosis affects about 10% of women in the reproductive age and is associated with infertility, dysmenorrhea, and chronic pelvic pain (McKinnon et al., 2018; Zhan et al., 2018a). Current treatments for endometriosis include surgery and pharmacotherapy (Ferrero et al., 2015; Ferrero et al., 2018). However, surgical approaches are sometimes inadequate (Horne et al., 2017). Pharmacotherapy uses GnRH agonist and progestin, and their effects on pain relief are relatively short termed with undesirable side effects limiting their prolonged use (Bedaiwy et al., 2017; Jensen et al., 2018). The exact pathogenesis of endometriosis is still unclear, and it is difficult to associate the pathogenesis of endometriosis to a single factor. Recent studies indicate that local inflammatory reaction in the peritoneal environment supports the development or maintenance of endometriosis, and the ectopic endometrial stromal cells (EESCs) are one of the major sources of cytokines (Khan et al., 2013; Takai et al., 2013; Zhan et al., 2018a).

Ahn et al. demonstrated that interleukin (IL)-17A was specifically elevated in the blood and endometrium of women with endometriosis, and its expression was reduced after surgical removal of lesions. Mechanistically, IL-17A was thought to contribute to the establishment and maintenance of endometriosis lesions by promoting angiogenesis and pro-inflammatory environment in the peritoneal cavity (Ahn et al., 2015). A critical regulator on chronic inflammation, serum IL-33, was abnormally elevated in women with endometriosis and principally in deeply infiltrating endometriosis. Elevated serum IL-33 was associated with the intensity of painful preoperative symptoms and the extent and severity of the deeply infiltrating endometriosis (Santulli et al., 2012). Furthermore, IL-33 contributed to the lesion survival and progression of endometriosis by perpetuating inflammation, angiogenesis, and lesion proliferation (Miller et al., 2017). These findings demonstrate inflammation to be a common factor involved in the regulation of endometriosis.

Nucleotide binding oligomerization domain-like receptors (NLRs) family caspase activation and recruitment domain containing 5 (NLRC5) is a novel sub-class of the NLR family. NLRC5 is a well-studied a transcriptional regulator of major histocompatibility complex (MHC) class I genes in immune cells and shuttles between the cytoplasm and the nucleus to play a leading role in the modulation of inflammatory response (Kobayashi and van den Elsen, 2012; Chelbi et al., 2017; Luan et al., 2018). Autophagy refers to a lysosome-dependent pathway by which cytoplasmic components are delivered to lysosome for degradation (Leidal et al., 2018). Over the years, accumulating evidence showed that autophagy was critically implicated in the progression of endometriosis (Mei et al., 2015; Zhan et al., 2018a; Liu et al., 2019). It was further indicated that autophagy was implicated in NLR-mediated inflammation. For example, ATG16L1 was shown to be crucial for the cytokine responses by nucleotide binding oligomerization domain (NOD) and the disruption of NOD1- or NOD2-ATG16L1 signaling axis-mediated pro-inflammation in Crohn’s disease (Travassos et al., 2010).

Lately, we found the level of NLRC5 was up-regulated in the endometrium with endometriosis compared to the endometrium with leiomyoma, implying the function of NLRC5 in the progression of endometriosis. Autophagy was down-regulated in the endometrium with endometriosis compared to that in endometrium with leiomyoma. Moreover, there exists negative correlation between NLRC5 and autophagy (Zhan et al., 2018b). Nevertheless, the exact molecular mechanism involving NLRC5 and autophagy in endometriosis is unknown. We hypothesizes that autophagy is implicated in NLRC5-mediated inflammation in ovarian endometriosis. In our present study, we evaluated the effects of NLRC5 in inflammation of EESCs. Specifically, we determined whether autophagy was involved in NLRC5-mediated inflammation in EESCs.

## Materials and Methods

### Ethics Statement

Ethics was approved for this study by the Institutional Review Board (IRB) of the Anhui Medical University. Human ectopic, eutopic endometrial tissue samples from endometriosis patients and control samples from leiomyoma women were collected as per institutional approved protocols and guidelines. Written informed consent was acquired before patient sample collection and storage. All methods were performed as per institutional approved guidelines.

### Subjects

The subjects recruited for the study were women of reproductive age attending the Department of Gynecology and Obstetrics in The Second Affiliated Hospital of Anhui Medical University between January 2017 and December 2018. The inclusion and exclusion criteria and sample collection methods used in this study were described in detail previously (Zhan et al., 2018b). Secretory phase ectopic endometrium and eutopic endometrium were acquired from 40 patients with ovarian endometriosis. The average age of the endometriosis patients was 35.8 ± 6.6 years. The staging and morphological distribution of lesions of endometriosis were based on the revised classification of the American Society for Reproductive Medicine (rASRM) (Revised American Society for Reproductive Medicine classification of endometriosis: 1996, 1997). Endometrium tissues obtain from women with leiomyoma are usually identified as normal endometrium tissues and used as control endometrium in previous studies (Choi et al., 2013). Control secretory phase normal endometrium tissues were collected at hysterectomy from five premenopausal women with leiomyoma. All the leiomyoma women have not taken any medications or received hormonal therapy at least 6 months prior to surgery. The average age of the participants from whom leiomyoma endometrial tissues were acquired was 37.9 ± 8.4 years. The phase of the menstrual cycle was validated by assessing the endometrial histology and by comparing the date to the expected day of the menstrual cycle provided by the patients. All collected endometrium samples were prepared for subsequent study.

### Isolation and Culture of Human ESCs/EESCs

Human endometrial tissues from endometriosis and leiomyoma patients were collected under sterile conditions and kept in Dulbecco’s modified Eagle’s medium (DMEM)/F12 (Invitrogen, USA) with an ice box. The tissues were processed within 3 h of collection. The tissues were rinsed with PBS and were minced into approximately 1 mm ×1 mm × 1 mm in size, and the appropriate amount of type I collagenase (Sigma, USA) was added. The mixture small pieces endometrial tissues were digested in a cell incubator at 5% CO_2_ 37°C for 60–80min. Filtration was performed with 200-mesh (74 μm aperture) and 400-mesh (38 μm pore size) wire sieve filters to remove debris and separate human EESCs. The filtrate was centrifuged at 800g for 5 min and then suspended in DMEM/F12 medium (Invitrogen, USA) supplemented with 12% fetal bovine serum (HyClone, Logan, UT, USA), 50 µg/ml of penicillin, and 50 µg/ml of streptomycin (Invitrogen, USA). The cells were transferred to a 25 cm^2^ culture bottle (Corning, USA) and cultured in an incubator with 5% CO_2_ at 37°C. All cultured EESCs undergo all following experimental procedures after the third passage. Immunofluorescence assay was carried out to detect EESCs marker vimentin (Liu et al., 2019). The vimentin antibody (ab92547, Abcam, Cambridge, MA, USA) was used as primary antibodies, and the second antibody was labeled green (A11037, Invitrogen, USA). 6-diamidine-2-phenylindole (DAPI) was used for nuclear staining. Pictures were taken under a fluorescence microscope (Nikon, Japan).

### RNA Extraction and Quantitative Real-Time PCR (qRT-PCR)

Total RNA was collected from cultured cells with the use of TRIzol reagent (Takara, Japan) following the manufacturer’s instructions. First-strand cDNA was synthesized using Thermoscript reverse transcription-polymerase chain reaction (RT-PCR) synthesis kit (Fermentas, USA). qRT-PCR analyses for mRNA of NLRC5, LC3, Beclin1, IL-6, TNF-α, and β-actin were carried out by using Thermoscript qRT-PCR kits (Fermentas, USA) in an ABI Prizm step-one plus real time PCR System (Applied Biosystems, USA). mRNA level of β-actin was used as an internal control. Relative expression levels were calculated based on the standard 2^-ΔΔCt^ method. All experiments were performed in triplicate and repeated at least three times. qRT-PCR primers are as given as in (**Table 1**).

**Table 1:** NLRC5, LC3, Beclin1, p62, IL-6, TNF-α, and β-actin primers for qRT-PCR.

Gene	Forward primers	Reverse primers
NLRC5	5’-GTTCTTAGGGTTCCGTCAGCG-3’	5’-CAGTCCTTCAGAGTGGCACAGAG-3’
LC3	5’-AGCAGCATCCAACCAAAATC -3’	5’-CTGTGTCCGTTCACCAACAG-3’
Beclin1	5’-AGCACCATGCAGGTGAGCTT-3’	5’-TGACACGGTCCAGGATCTTG-3’
p62	5’-CTGCTGCCTCCCTCTAATCC-3’	5’-TATTCTCCGGCTCCATCTTG-3’
IL-6	5’-CCTGACCCAACCACAAATGC-3’	5’-ATCTGAGGTGCCCATGCTAC-3’
TNF-α	5’-CCCCAGGGACCTCTCTCTAATC-3’	5’-GGTTTGCTACAACATGGGCTACA-3’
β-actin	5’-CACCCAGCACAATGAAGATCAAGAT-3’	5’-CCAGTTTTTAAATCCTGAGTCAAGC-3’

### Protein Extraction and Western Blotting Analysis

Human endometrial tissues and EESCs were lysed in protein extraction solution (Beyotime, China). Protein concentration was calculated by the BCA assay kit (SinoBio Biotech, China). Cell lysates collected by centrifugation and transferred onto polyvinylidene difluoride (PVDF) membrane (Millipore Corp, Billerica, MA, USA). After membranes were blocked, nitrocellulose blots were incubated for 6 h with primary antibodies diluted in primary antibody dilution buffer (Beyotime, China). The primary antibodies recognizing NLRC5 (ab105411, Abcam, Cambridge, MA, USA), LC3 (ab192890, Abcam, Cambridge, MA, USA), Beclin1 (ab207612, Abcam, Cambridge, MA, USA), p62 (18420-1-AP, Proteintech), IL-6 (ab6672, Abcam, Cambridge, MA, USA), TNF-α (ab6671, Abcam, Cambridge, MA, USA), and β-actin (GB12001, Servicebio, Wuhan, China) were used at 1:1,000, 1:1,000, 1:1,000, 1:1,000, 1:1,000, 1:1,000, 1:1,000, and 1:3000, respectively. The membranes were then incubated in TBST containing 5% skim milk at 37°C for 4 h and with specific primary antibodies at 4°C overnight. Then, the membranes were washed with TBS/Tween20 for three times, followed by incubation with horseradish peroxidase (HRP)-conjugated secondary antibodies (1:10,000) at 37°C for 1 h. After washing three times with TBST (Boster, China), proteins were visualized with ECL chemiluminescent kit (ECL-plus, Thermo Scientific, USA). We measured protein levels of LC3-II, which represents the protein levels of LC3. All experiments were performed in triplicate and repeated at least three times.

### EESCs Transient Transfection With NLRC5 Plasmid and siRNA-NLRC5

The full-length coding region of NLRC5 was created from human genomic DNA by RT-PCR using the primers in (**Table 2**). The PCR products were cloned into the pEGFP-C2 empty vector by using EcoR I/BamH I. The recombinant construct pEGFP-C2-NLRC5 was verified by direct DNA sequencing. Small interfering RNA (siRNA) oligonucleotides against NLRC5 or scrambled sequences were designed and synthesized by the Gema Pharma Corporation (Shanghai, China) and contained the sequences in (**Table 3**). EESCs were subjected to transfection with constructed plasmid and siRNA using Lipofectamine™ 2000 (Invitrogen, USA) according to the manufacturer’s protocol.

**Table 2:** NLRC5 plasmid primers.

Gene	Forward primer	Reverse primer
NLRC5 plasmid	5’’-CCGGAATTCCGGATGGCCAGGAAGCTGGA-3’	5’-GGGATCCCGTCACCTGAGTGTCTTCCCA-3’

**Table 3:** siRNA-NLRC5 and scrambled-RNAi plasmid sequences.

Gene	Sense	Antisense
SiRNA-NLRC5	AAGAACGAGAGACUCUGCCAACUGCdTdT	GCAGUUGGCAGAGUCUCUCGUUCUUdTdT
Scrambled-RNAi	UUCUCCGAACGUGUCACGUTT	ACGUGACACGUUCGGAGAATT

### EESCs Treatment With Autophagy Inhibitor Chloroquine (CQ) and Aautophagy Agonist Rapamycin

CQ (Sigma, USA) and rapamycin (Sigma, USA) were dissolved in dimethyl sulfoxide (DMSO, Sigma, USA) and used at a concentration of 30 μM and 1 nM, respectively. EESCs were seeded overnight in culture dishes and transfection with NLRC5 plasmid, 6 h later, EESCs treated with CQ and rapamycin for 48 h.

### ELISA for IL-6 and TNF-α

The expressions of IL-6 and TNF-α in the supernatants of each EESCs culture were validated by using the enzyme-linked immune sorbent assay (ELISA) kit (R&D, USA) according to the manufacturer’s instruction. Samples were run in duplicates. Inter-assay coefficients of variation were calculated with the results obtained in 10 different assays performed at different time using different plates (R&D Systems, USA). Intra-assay coefficients of variation were calculated for 10 replicates of the sample in the same plate (R&D Systems, USA).

### Immunofluorescence for Location of NLRC5, LC3, and Beclin1 in Human EESCs

Human EESCs were washed with PBS and fixed in 4% paraformaldehyde. Nonspecific binding sites were incubated with 0.1% bovine serum albumin in PBS, and the fixed cells were then stained with, anti-NLRC5, anti-LC3, anti-Beclin1 antibodies (1:200) in PBS at room temperature. After this, cells were incubated with Alexa Fluor 488-conjugated and 568-conjugated secondary antibodies (1:5,000, Vector Laboratories, Burlingame, CA, USA) at dark room. The slides were mounted in mounting medium, and nuclei were counter stained with 4′,6-diamidino-2-phenylindole at room temperature. Images were captured using a laserscanning confocal microscope (Nikon, Japan).

### Autophagy Flow Detection Using GFP-RFP-LC3 Adenovirus Vector

EESCs were seeded on coverslips in 12-well plates and allowed to reach 50–70% confluence at the time of transfection. The GFP-RFP-LC3 adenovirus construct were purchased from Shanghai Genechem Co. Ltd. Adenovirus was infected into the cells according to the manufacturer’s protocol. EESCs were incubated in growth medium with the adenoviruses at a multiplicity of infection (MOI) of 100 for 24 h to ensure the expression of GFP-RFP-LC3. Autophagy was observed using a laser scanning confocal microscope (Nikon, Japan). Autophagic flux was determined by evaluating the number of the yellow puncta, which represented autophagosomes. Quantification of mean yellow puncta of 10–15 cells per condition using Image J software.

### Transmission Electron Microscopy (TEM)

EESCs were fixed with 2.5% glutaraldehyde in 0.1 M cacodylate buffer (pH 7.4) for 45 min at 4°C, then washed in cacodylate buffer, post-fixed in 1% OsO4 in cacodylate buffer for 3 h, then dehydrated at 25°C with a graded series of ethanol, and embedded in Eponate. Ultra-thin sections were subjected to double staining with uranyl acetate, and images were then captured with a transmission electron microscope (Hitachi 7700; Hitachi High Technologies).

### Statistical Analysis

All data were analyzed by SPSS 23.0 software (Chicago, USA). Data were expressed as mean ± standard error of measurement (SEM). Statistical analysis was performed using analysis of variance (ANOVA). Statistically significant differences between the treatment groups were identified using Duncan’s multiple range test. P < 0.05 was considered statistically significant.

## Results

### NLRC5 and Inflammation Are Up-Regulated, and Autophagy Is Down-Regulated in Endometrial Stromal Cells (ESCs) of Ovarian Endometriosis Patients With Endometriosis Compared to ESCs of Patients With Leiomyoma

**Figure 1:**
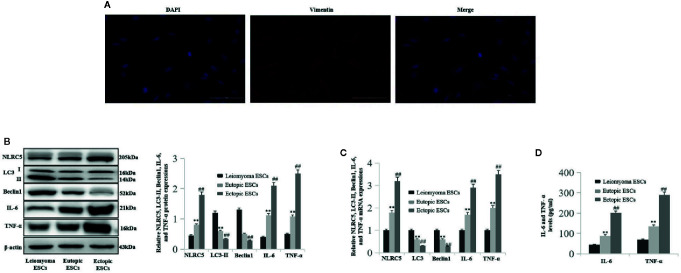
The levels of NLRC5, inflammation, and autophagy in ESCs of patients with endometriosis (n = 5) and patients with leiomyoma (n = 5). **(A)** Representative image of immunofluorescence staining showing human ESCs displayed long-spindle with positively expressing vimentin and negatively expressing keratin. Photographs were taken at magnifications of 400×. **(B**, **C)** Representative western blotting and qRT-PCR showing NLRC5, IL-6, and TNF-α were up-regulated in endometriosis ectopic and eutopic ESCs of patients with endometriosis compared to the ESCs of patients with leiomyoma (^**^P < 0.01 vs. leiomyoma ESCs), and the levels of NLRC5, IL-6, and TNF-α in ectopic ESCs were also significantly higher than in the eutopic ESCs (^##^P < 0.01 vs. eutopic ESCs). Autophagy-related molecules LC3 and Beclin1 were down-regulated in ectopic and eutopic ESCs of patients with endometriosis compared to the ESCs of patients with leiomyoma (^**^P < 0.01 vs. leiomyoma ESCs), and the expression of LC3 and Beclin1 in ectopic ESCs was also significantly lower than in the eutopic ESCs (^##^P < 0.01 vs. eutopic ESCs). **(D)** Representative ELISA showing IL-6 and TNF-α were up-regulated in endometriosis ectopic and eutopic ESCs of patients with endometriosis compared to the ESCs of patients with leiomyoma (^**^P < 0.01 vs. leiomyoma ESCs), and the levels of IL-6 and TNF-α in ectopic ESCs were also significantly higher than in the eutopic ESCs (^##^P < 0.01 vs. eutopic ESCs). The expression levels of mRNA were normalized with respect to β-actin and were calculated using the 2^-ΔΔCt^ method. The protein expression levels were quantified by Image J software and normalized to β-actin protein levels. The results are represented as the mean ± SEM from at least three independent experiments.

To investigate the levels of NLRC5 inflammation and autophagy in ESCs, we first identified primary human ESCs with immunofluorescence. Vimentin is the specific marker of human ESCs. As shown in **Figure 1A**, human ESCs displayed long spindle with positively expressing vimentin. Western blotting and qRT-PCR were performed to compare the levels of NLRC5 inflammation and autophagy in ESCs of patients with endometriosis and ESCs of patients with leiomyoma. As shown in **Figures 1B–D**), NLRC5, IL-6, and TNF-α were up-regulated in ectopic and eutopic ESCs of patients with endometriosis compared to that in the ESCs of patients with leiomyoma, and the levels of NLRC5, IL-6, and TNF-α in ectopic ESCs were also significantly higher than that in eutopic ESCs. Expression of autophagy-related molecules LC3 and Beclin1 was down-regulated in ectopic and eutopic ESCs of patients with endometriosis compared to that in the ESCs of patients with leiomyoma, and the expression of LC3 and Beclin1 in ectopic ESCs was also significantly lower than that in eutopic ESCs.

### Over-Expression of NLRC5 Enhances Autophagy Induction in EESCs

**Figure 2:**
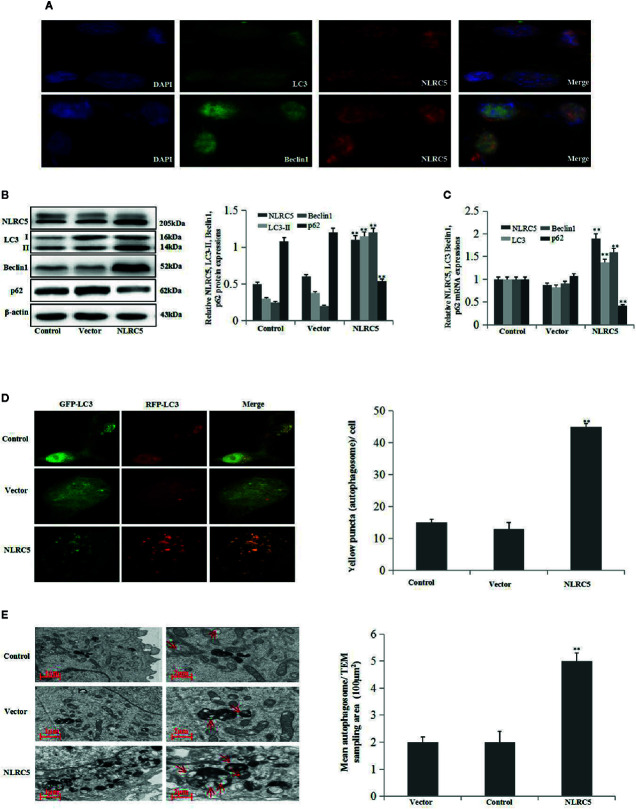
Effect of NLRC5 over-expression on autophagy in EESCs (n = 7). **(A)** Representative image of immunofluorescence staining showing NLRC5 and LC3, Beclin1 are present both in the cytoplasm and nucleus, NLRC5 and LC3, Beclin1 were co-located in the nucleus. Photographs were taken at magnifications of 1600×. **(B**, **C)** Representative western blotting and qRT-PCR results showing over-expression of NLRC5 by transfection with NLRC5 plasmid significantly promoted NLRC5, LC3 Beclin1 expressions, and inhibited p62 expression when compared with vector group (^**^P < 0.01 vs. vector group). **(D)** Representative images showing LC3 staining in EESCs infected with GFP-RFP-LC3 adenovirus, over-expression of NLRC5 by transfection with NLRC5 plasmid significantly promoted yellow puncta when compared with vector group. Photographs were taken at magnifications of 1,600×, quantification of mean yellow puncta of 10–15 cells per condition is shown (^**^P < 0.01 vs. vector group). **(E)** Representative transmission electron microscopy (TEM) showing over-expression of NLRC5 by transfection with NLRC5 plasmid significantly promoted autophagosomes formation when compared with vector group, autophagosomes were highlighted by red arrows (left scale bar: 1μm; right scale bar: 2μm; ^**^P < 0.01 vs. vector group). The expression levels of mRNA were normalized with respect to β-actin and were calculated using the 2^-ΔΔCt^ method. The protein expression levels were quantified by Image J software and normalized to β-actin protein levels. The results are represented as the mean ± SEM from at least three independent experiments.

To determine whether NLRC5 was implicated in the control of autophagy in ovarian endometriosis, immunofluorescence staining was first performed to examine the sub-cellular localization of NLRC5, LC3, and Beclin1 in EESCs. As shown in **Figure 2A**, NLRC5, LC3, and Beclin1 were localized to both cytoplasm and nucleus. Furthermore, NLRC5, LC3, and Beclin1 were co-localized in the nucleus. As shown in **Figures 2B, C** by western blotting and qRT-PCR analyses, over-expression of NLRC5 by transfection with NLRC5 plasmid significantly induced NLRC5, LC3, and Beclin1 expressions and inhibited p62 expression compared with vector group, indicating that over-expression of NLRC5 contributed to autophagy induction in EESCs. To further confirm if over-expression of NLRC5 can induce autophagy, we utilized the tandem GFP-RFP-LC3 adenovirus construct. As shown in **Figure 2D**, more yellow puncta were presented in NLRC5 plasmid treated EESCs than in the vector treated EESCs. TEM is one of the most reliable methods for the observation of autophagy and quantification of autophagic accumulation. As shown in **Figure 2E**, over-expression of NLRC5 increased the number of autophagosomes compared with vector group in EESCs.

### Inhibition of NLRC5 Restricts Autophagy Induction in EESCs

**Figure 3:**
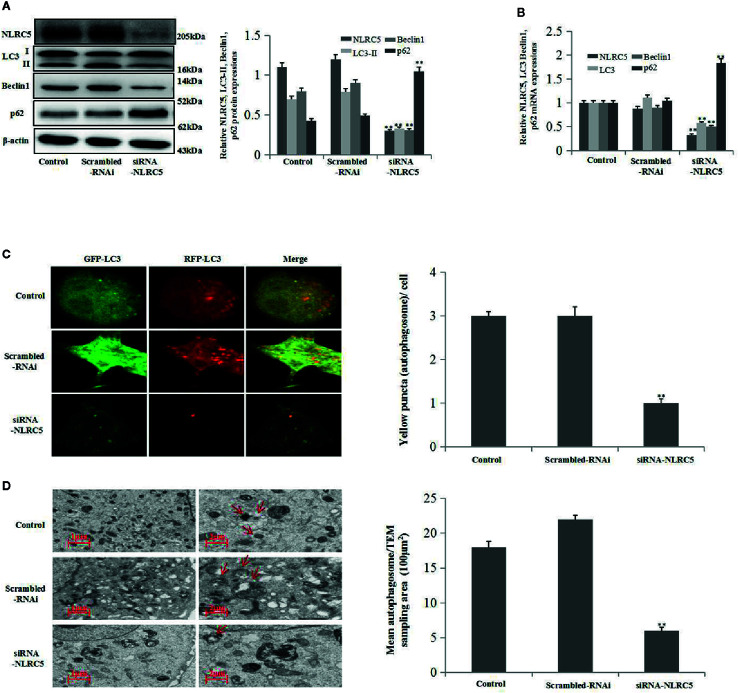
Effects of NLRC5 inhibition on autophagy in EESCs (n = 7). **(A**, **B)** Representative western blotting and qRT-PCR results showing inhibition of NLRC5 by transfection with siRNA-NLRC5 significantly inhibited NLRC5, LC3 Beclin1 expressions, and promoted p62 expression when compared with those from the scrambled-RNAi group (^**^P < 0.01 vs. scrambled-RNAi group). **(C)** Representative images showing LC3 staining in EESCs infected with GFP-RFP-LC3 adenovirus; inhibition of NLRC5 by siRNA-NLRC5 transfection significantly inhibited yellow puncta when compared with scrambled-RNAi group. Photographs were taken at magnifications of 1600×, quantification of mean yellow puncta of 10-15 cells per condition is shown (^**^P < 0.01 vs. scrambled-RNAi group). **(D)** Representative TEM image showing inhibition of NLRC5 by siRNA-NLRC5 transfection significantly inhibited autophagosomes formation when compared with scrambled-RNAi group, autophagosomes were highlighted by red arrows(left scale bar: 1μm; right scale bar: 2μm; ^**^P < 0.01 vs. scrambled-RNAi group). The expression levels of mRNA were normalized with respect to β-actin and were calculated using the 2^-ΔΔCt^ method. The protein expression levels were quantified by Image J software and normalized to β-actin protein levels. The results are represented as the mean ± SEM from at least three independent experiments.

To further establish the role of NLRC5 in autophagy, we inhibited the expression of NLRC5 by transfecting EESCs with siRNA-NLRC5. As shown in **Figures 3A, B** by western blotting and qRT-PCR analyses, NLRC5-siRNA significantly inhibited NLRC5, LC3, and Beclin1 expressions and promoted p62 expression compared with scrambled-RNAi group. Furthermore, **Figure 3C** depicted a decrease in yellow puncta in siRNA-NLRC5 treated EESCs than in scrambled-RNAi treated EESCs. In addition, as shown in **Figure 3D** by TEM analysis, inhibition of NLRC5 decreased the number of autophagosomes compared with scrambled-RNAi group in EESCs.

### CQ Decreases the Role of NLRC5 in Autophagy

**Figure 4:**
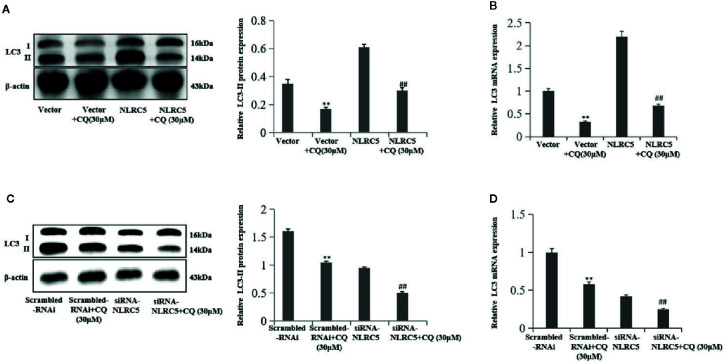
CQ decreases the role of NLRC5 in autophagy (n = 5). **(A**, **B)** Representative western blotting and qRT-PCR results showing treatment with 30μM autophagy inhibitor CQ restricted the level of LC3-II in NLRC5-overexpressed EESCs compared with NLRC5 plasmid group (^**^P < 0.01 vs. vector group and ^##^P < 0.01 vs. NLRC5 group). **(C**, **D)** Representative western blotting and qRT-PCR results showing treatment with 30μM autophagy inhibitor CQ inhibited the level of LC3-II in NLRC5-down-regulated EESCs compared with siRNA-NLRC5 group (^**^P < 0.01 vs. scrambled-RNAi group and ^##^P < 0.01 vs. siRNA-NLRC5 group).The expression levels of mRNA were normalized with respect to β-actin and were calculated using the 2^-ΔΔCt^ method. The protein expression levels were quantified by Image J software and normalized to β-actin protein levels. The results are represented as the mean ± SEM from at least three independent experiments.

We further confirmed the role of NLRC5 in autophagy by using autophagy inhibitor CQ to inhibit autophagy. We use 30 μM CQ to inhibit autophagy. As shown in **Figures 4A, B** by western blotting and qRT-PCR analyses, treatment with CQ restricted the level of LC3-II in NLRC5-overexpressed EESCs compared with NLRC5 plasmid group. And as shown in **Figures 4C, D** by western blotting and qRT-PCR analyses, treatment with CQ inhibited the level of LC3-II in siRNA-NLRC5 EESCs compared with siRNA-NLRC5 group.

### NLRC5 Inhibits Inflammation in EESCs

**Figure 5:**
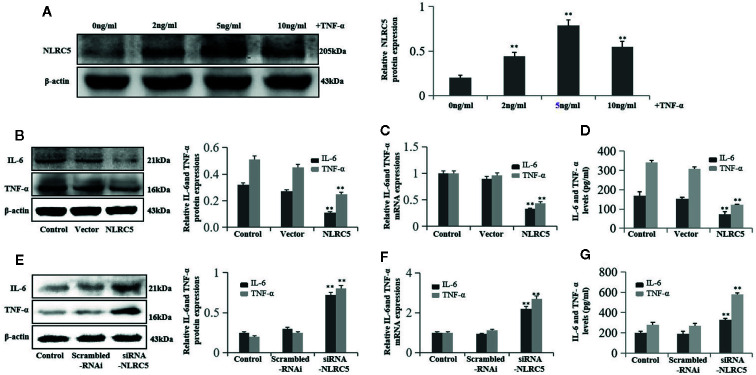
NLRC5 inhibits inflammation in EESCs (n = 7). **(A)** Representative western blotting showing 2, 5, and 10 ng/ml of TNF-α all induced NLRC5 expressions in EESCs compared with control group, and the highest protein levels of NLRC5 were reached at 5 ng/ml after 24 h induced by TNF-α treatment (^**^P < 0.01 vs. control group). **(B**–**D)** Representative western blotting, qRT-PCR, and ELISA showing over-expression of NLRC5 by transfection with NLRC5 plasmid significantly inhibited IL-6 and TNF-α expressions when compared with vector group (^**^P < 0.01 vs. vector group). **(E**–**G)** Representative western blotting, qRT-PCR, and ELISA showing inhibition of NLRC5 by transfection with siRNA-NLRC5 significantly promoted IL-6 and TNF-α expression when compared with scrambled-RNAi group (^**^P < 0.01 vs. scrambled-RNAi group). The expression levels of mRNA were normalized with respect to β-actin and were calculated using the 2^-ΔΔCt^ method. The protein expression levels were quantified by Image J software and normalized to β-actin protein levels. The results are represented as the mean ± SEM from at least three independent experiments.

To investigate the role of NLRC5 in inflammation in endometriosis, we first identified the role of TNF-α in NLRC5 in EESCs. As seen in **Figure 5A**, 2, 5, and 10 ng/ml TNF-α all induced NLRC5 expressions in EESCs compared with control group, and the highest protein levels of NLRC5 were reached at 5 ng/ml after 24 h induced by TNF-α treatment. The levels of inflammation were assessed by western blotting, qRT-PCR, and ELISA for IL-6 and TNF-α. Over-expression of NLRC5 by transfection with NLRC5 plasmid significantly inhibited IL-6 and TNF-α expression (**Figures 5B–D**), and inhibition of NLRC5 using siRNA-NLRC5 significantly induced IL-6 and TNF-α expression compared with vector group and scrambled-RNAi group (**Figures 5E–G**), respectively.

### NLRC5 Inhibits Inflammation by Promoting Autophagy in EESCs

**Figure 6:**
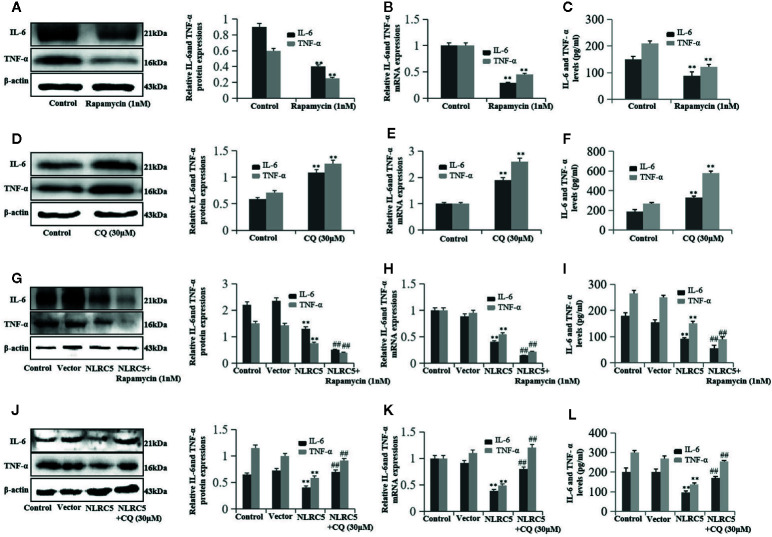
NLRC5 inhibits inflammation in EESCs by promoting autophagy (n = 9). **(A–C)** Representative western blotting, qRT-PCR, and ELISA showing promotion of autophagy by using 1 nM autophagy agonist rapamycin significantly inhibited IL-6 and TNF-α expression when compared with control group (^**^P < 0.01 vs. control group). **(D–F)** Representative western blotting, qRT-PCR, and ELISA showing inhibition of autophagy by using 30 μM autophagy inhibitor CQ significantly promoted IL-6 and TNF-α expression when compared with control group (^**^P < 0.01 vs. control group). **(G–I)** Representative western blotting, qRT-PCR, and ELISA showing promotion of autophagy by 1 nM rapamycin contributed to the NLRC5-mediated inhibition of IL-6 and TNF-α expression in EESCs (^**^P < 0.01 vs. vector group and ^##^P < 0.01 vs. NLRC5 group). **(G)** Representative western blotting, qRT-PCR, and ELISA showing inhibited autophagy by 30 μM CQ restricted the NLRC5-mediated inhibition of IL-6 and TNF-α expression in EESCs (^**^P < 0.01 vs. vector group and ^##^P < 0.01 vs. NLRC5 group). The expression levels of mRNA were normalized with respect to β-actin and were calculated using the 2^-ΔΔCt^ method. The protein expression levels were quantified by Image J software and normalized to β-actin protein levels. The results are represented as the mean ± SEM from at least three independent experiments.

First, we investigated the role of autophagy in inflammation in EESCs. We use 1 nM rapamycin and 30 μM CQ to promote and inhibit autophagy, respectively. As shown in **Figures 6A–C**, promotion of autophagy by rapamycin significantly inhibited IL-6 and TNF-α expression compared with control group; inhibition of autophagy by CQ significantly induced IL-6 and TNF-α expression compared with control group (**Figures 6D–F**). To further determine whether autophagy is involved in the regulation of NLRC5-mediated anti-inflammation, EESCs were transfected with NLRC5 plasmid and treated with rapamycin or CQ. **Figures 6G–I** depicted that promotion of autophagy contributed to the NLRC5-mediated inhibition of IL-6 and TNF-α expression compared with NLRC5 plasmid group in EESCs. **Figures 6J–L** indicated that inhibition of autophagy restricted the NLRC5-mediated inhibition of IL-6 and TNF-α expression compared with NLRC5 plasmid group in EESCs.

## Discussion

Pathogen-associated molecular patterns (PAMPs) provide the first line of defense against invading microbes in the innate immune response. Pattern recognition receptors (PRRs) are critical sensors in the PAMP recognition (Akira et al., 2006; Medzhitov, 2007). NLRs are a recently discovered family of cytoplasmic PRRs. NLRs are evolutionary conserved proteins that serve as sentinels for microbes to trigger induction of innate and adaptive immunity (Davis et al., 2011; Yuen et al., 2014). Recent evidence also showed that certain NLRs function beyond the innate immunity, such as in cancer and inflammation (Allen et al., 2012; Fekete et al., 2018; Wang et al., 2019). NLRC5 is a novel identified member of the NLR family (Meissner et al., 2010; Chelbi et al., 2017). Accumulating evidence from research presents that NLRC5 plays an important role in immune evasion of cancers, and is a therapeutic target. High levels of NLRC5 are associated with higher survival index (Yoshihama et al., 2016; Yoshihama et al., 2017). Furthermore, novel studies reveal that NLRC5 is up-regulated and implicated in tumorigenesis by promoting cell proliferation, migration, and invasion in high inflammatory state related cancer (He et al., 2016; Peng et al., 2016). Notably, studies propose that NLRC5 also plays a key role in inflammation, but the conclusion is controversial. For example, Cui et al. identify that NLRC5 inhibits NF-κB activation by binding to IKKα and IKKβ. Inhibiting NLRC5 rescues the activation of NF-κB and the inflammatory response in RAW264.7 cells (Cui et al., 2010). Using LX-2 cells, Xu and co-workers show that over-expression of NLRC5 leads to an up-regulation of IL-6 and IL-1β secretion (Xu et al., 2015).

Endometriosis is a chronic disease which affecting about 10% of women of reproductive age. Endometriotic lesions are considered to be nonmalignant lesions that are defined as the ectopic presence of endometrial glands and stroma outside of the uterus. However, endometriosis also represents some features of malignant neoplasms, such as local invasion and resistance to apoptosis (Bulun, 2009; Anglesio et al., 2017). For example, it has been demonstrated that endometriosis is intimately involved in an estimated incidence of 0.72% and is limited to clear cell endometrioid and low-grade serous tumors (Pearce et al., 2012). It has been demonstrated that the high inflammatory level in the peritoneal environment contributes to the pathogenesis of endometriosis, the EESCs are one of the major sources of cytokines (Khan et al., 2013; Takai et al., 2013; Zhan et al., 2018a). In our previous study employing immunohistochemistry, we observe that NLRC5 is up-regulated in ectopic and eutopic endometrium of patients with endometriosis compared to the endometrium of patients with leiomyoma. Furthermore, the level of NLRC5 in ectopic endometrium is also obviously higher than in the eutopic endometrium, which reaffirms that NLRC5 may have a close relationship the development of endometriosis (Zhan et al., 2018b). In our present study, we find the expressions of NLRC5, IL-6, and TNF-α in ectopic and eutopic ESCs of patients with endometriosis are up-regulated compared to the ESCs of patients with leiomyoma, and the levels of NLRC5, IL-6, and TNF-α in ectopic ESCs are also obviously higher than in the eutopic ESCs. Furthermore, NLRC5 is induced by TNF-α n the EESCs, and over-expression of NLRC5 results in the down-regulation of IL-6 and IL-21β secretion. Knockdown of NLRC5 by siRNA induces IL-6 and IL-1β secretion. These results suggest that, like in high inflammatory state related cancer, NLRC5 was up-regulated in endometriosis owing to its inflammatory state. Furthermore, NLRC5 acts as an anti-inflammation regulator in ovarian endometriosis.

Autophagy is an evolutionarily conserved mechanism involving clearance of damaged cellular components to facilitate recycling and degradation of cytoplasmic constituents. Autophagic response contributes to overcoming toxic or damaged products or to maintain cellular homeostasis (Boga et al., 2019; Clarke and Simon, 2019). It is now established that autophagy plays dual roles in cell growth and cell death in human diseases and physiology. Activation or blockage of autophagy has been proposed as a promising approach to improve human health (Mizushima et al., 2008; Jiang et al., 2015). A growing body of research presents a potential evidence that autophagy is crucial for the progression of endometriosis. However, seemingly opposing conclusion with respect to the function of autophagy in endometriosis has been proposed, with controversial evidence concerning the expression levels of autophagy markers in endometriosis. Liu and co-workers show that the expression of LC3 in the ectopic endometrium is visibly higher than that in normal endometrium and eutopic endometrium from women with endometriosis (Liu et al., 2018). On the contrary, Mei et al. suggest that the levels of autophagy-related molecules LC3 and Beclin1 in ectopic ESCs are significantly decreased compare to those in normal ESCs (Mei et al., 2018). Interestingly, NLRs have recently been crucially associated with the control of autophagy. For example, NLRX1 interacts with Beclin1, which is shown to be responsible for the NLRX1-mediated inhibition of invasion and autophagic processes during Group A *Streptococcus* (GAS) infection (Aikawa et al., 2018). Jin et al. indicate that deficient of NLRP3 augments neutrophils survival by decreasing autophagy and enhancing phagocytosis during polymicrobial sepsis (Jin et al., 2017). However, whether NLRC5 is implicated in the control of autophagy remains unclear. In our study, we find that autophagy-related molecules LC3 and Beclin1 are down-regulated in ectopic and eutopic ESCs of patients with endometriosis compare to the ESCs of patients with leiomyoma, and the expression of LC3 and Beclin1 in ectopic ESCs is also significantly lower than in the eutopic ESCs. NLRC5 co-localized with LC3 and Beclin1 in nucleus in EESCs. Importantly, over-expression of NLRC5 contributes towards the promotion of autophagy in EESCs. In line with this observation, inhibition of NLRC5 leads to the down-regulation of autophagy in EESCs. These results indicate the positive role of NLRC5 in autophagy. Nevertheless, the potential underlying mechanisms between NLRC5 and autophagy is not clear. Travassos suggests that the sub-classes of the NLRs, NOD1, and NOD2 are essential for anti-bacterial infection by recruiting autophagy protein ATG16L1 to the plasma membrane at the bacterial entry site (Travassos et al., 2009). Recently, Xi and colleagues indicate that NOD1 has a dark side in hepatic ischemia-reperfusion injury by activating autophagy protein ATG5 (Xi et al., 2019). In view of NLRC5 co-localized with LC3 and Beclin1 in nucleus in EESCs from patients with endometriosis, furthermore, NLRC5 led to the expressions of LC3 and Beclin1, we speculate that NLRC5 may promote autophagy by activating autophagy protein Beclin1 or other ATGs, which need to be validated in the future.

**Figure 7:**
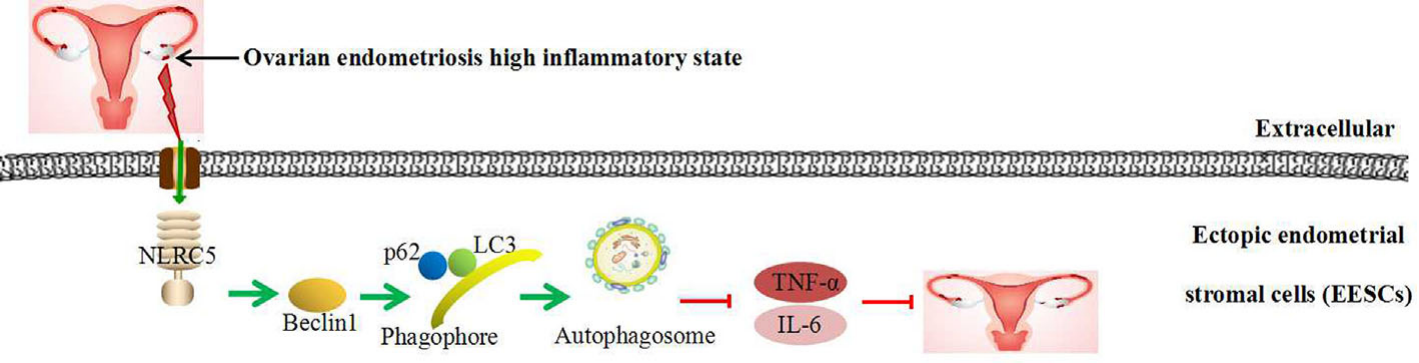
Schematic diagram of NLRC5-regulated autophagy in EESCs of ovarian endometriosis.

Recent evidence depicts the involvement of autophagy in inflammatory reactions. Our results reveal that autophagy plays a negative role in inflammation in EESCs. In addition, promotion of autophagy using rapamycin contributes to NLRC5-mediated anti-inflammation and inhibition of autophagy using CQ prevents NLRC5-mediated anti-inflammation in EESCs. Collectively, although our previous study suggests that there is a negative correlation between NLRC5 and autophagy in endometriosis. Our present findings demonstrate that inflammatory state in ectopic endometrium of ovarian endometriosis activates NLRC5. NLRC5 acts as an inhibitor of inflammation in ovarian endometriosis by inducing autophagy: Over-expression of NLRC5 could promote the expressions of LC3, Beclin1, and autophagosomes formation in EESCs, thereby inhibiting IL-6 and TNF-α expressions in ovarian endometriosis, suggesting that promoting NLRC5 and autophagy may be novel therapeutic methods in ovarian endometriosis (**Figure 7**). This study may provide a possible explanation that if inflammation-activated NLRC5 was not sufficient, then ovarian endometriosis could grow rapidly *via* inhibiting autophagy. When NLRC5 is sufficiently promoted in EESCs, NLRC5 could inhibit inflammation by positively regulate expression of autophagy markers.

Although our results are interesting, we are aware that our study comes up some limitations: Due to the limitations of obtaining surgical specimens, ectopic endometrial tissues were collected all from women diagnosed with ovarian endometriosis, and the underlying mechanism of how NLRC5 regulates autophagy is undefined; we should use more experiment methods and included more inflammatory biomarkers to detect the inflammation levels; no animal experiments have been conducted in our study.

## Data Availability Statement

The raw data supporting the conclusions of this article will be made available by the authors, without undue reservation, to any qualified researcher.

## Ethics Statement

The studies involving human participants were reviewed and approved by The institutional review board (IRB) of the Anhui Medical University. The patients/participants provided their written informed consent to participate in this study.

## Author Contributions 

LZ conceived the experiments; ZZW collected patient samples; RHH, SYS, and LTF conducted the experiments. WYW, YJF, and LJS analyzed the results; RHH wrote the manuscript; LZ and YXC supervised data analysis and provided critical suggestions for manuscript writing. XJL, JZ, and YXC revised the manuscript. All authors contributed to the article and approved the submitted version.

## Funding

The project was supported by the by the Natural Science Foundation of Colleges and Universities (KJ2017A197), the National Science Foundation of China (81802586), the Special Funds for the Development of Science and Technology of Anhui Province (YDZX20183400004194), the Research Fund Project granted from Anhui Research Institute of Translational Medicine (2017zhyx30), and the 2018 Anhui Key Research and Development Project (1804a07020128).

## Conflict of Interest

The authors declare that the research was conducted in the absence of any commercial or financial relationships that could be construed as a potential conflict of interest.
